# Toward Benchmarking of Long-Term Spatio-Temporal Maps of Pedestrian Flows for Human-Aware Navigation

**DOI:** 10.3389/frobt.2022.890013

**Published:** 2022-07-04

**Authors:** Tomáš Vintr, Jan Blaha, Martin Rektoris, Jiří Ulrich, Tomáš Rouček, George Broughton, Zhi Yan, Tomáš Krajník

**Affiliations:** ^1^ Laboratory of Chronorobotics, Artificial Intelligence Center, Department of Computer Science, Faculty of Electrical Engineering, Czech Technical University in Prague, Prague, Czech Republic; ^2^ CIAD UMR 7533, Univ. Bourgogne Franche-Comté, UTBM, Montbéliard, France

**Keywords:** long-term navigation, planning, spatio-temporal modeling, human-aware navigation, scheduling, pedestrian flows

## Abstract

Despite the advances in mobile robotics, the introduction of autonomous robots in human-populated environments is rather slow. One of the fundamental reasons is the acceptance of robots by people directly affected by a robot’s presence. Understanding human behavior and dynamics is essential for planning when and how robots should traverse busy environments without disrupting people’s natural motion and causing irritation. Research has exploited various techniques to build spatio-temporal representations of people’s presence and flows and compared their applicability to plan optimal paths in the future. Many comparisons of how dynamic map-building techniques show how one method compares on a dataset versus another, but without consistent datasets and high-quality comparison metrics, it is difficult to assess how these various methods compare as a whole and in specific tasks. This article proposes a methodology for creating high-quality criteria with interpretable results for comparing long-term spatio-temporal representations for human-aware path planning and human-aware navigation scheduling. Two criteria derived from the methodology are then applied to compare the representations built by the techniques found in the literature. The approaches are compared on a real-world, long-term dataset, and the conception is validated in a field experiment on a robotic platform deployed in a human-populated environment. Our results indicate that continuous spatio-temporal methods independently modeling spatial and temporal phenomena outperformed other modeling approaches. Our results provide a baseline for future work to compare a wide range of methods employed for long-term navigation and provide researchers with an understanding of how these various methods compare in various scenarios.

## 1 Introduction

The last decade showed that the advances in the robotics field enable autonomous robots to operate in human-populated environments (Triebel et al., 2016; Hawes et al., 2017; Coşar et al., 2020). The human-populated environment includes diverse types of dynamics, such as natural daily (Krajnik et al., 2014a) and seasonal changes (Neubert et al., 2013) but, most importantly, the dynamics imposed by human actions (Tipaldi et al., 2013). The robots that take these dynamics into consideration and adjust their decisions provide better performance of their human-centric tasks (Santos et al., 2016, 2017; Hanheide et al., 2017; Hawes et al., 2017; Kunze et al., 2018; Calderita et al., 2021). Moreover, later works assert that decisions of autonomous robots that consider human habits are crucial for robots to be accepted by human society (Triebel et al., 2016; Palmieri et al., 2017; Vintr et al., 2020). Based on the principles of modeling the human-imposed dynamics in aforementioned long-term experiments, we believe that human customs and behavior are deeply connected to the abstract structure of the time represented as a calendar and clock. Furthermore, we claim the robots that are supposed to be part of human society need to accept this structure, similar to their acceptance of the spatial structures like walls.

We state that the long-term human-aware navigation needs to include spatio-temporal maps into the navigation system to support the decisions in advance of the navigational task. The traditional approach to robot navigation in an uncontrolled environment is using a combination of static maps (Elfes, 1989; Cadena et al., 2016) with a reactive approach to unexpected events. However, many unexpected events are usually caused by human actions, especially human movement through the robot’s operational environment. The reactive replanning of a robot’s trajectory based on sense-plan-act frameworks, used, for example, in the Robot Operating System (Quigley et al., 2009) move base, gives people an impression of clumsiness (Hebesberger et al., 2017) due to their slow response, which eventually leads to negative emotions toward the robot (Vintr et al., 2020). This unwanted interaction is mainly driven by the fact that by the time the robot can detect people walking around it and replan its trajectories, the person is already taking evasive action. Therefore, incorporating the expected human movement through the environment into the robot’s navigation and planning is of high importance for the autonomous robots intended to help people in their environment (Krajník et al., 2020; Rudenko et al., 2020; Palmieri et al., 2021). To show the fundamental impact on human acceptance when incorporating spatio-temporal maps into the navigation, we provide a field robot experiment with an industrial-grade robotic platform. The experiment studied how many people traversing the university hall were irritated by the robot planning its navigation with and without the map.

As the spatio-temporal maps were used in different scientific fields, we created a comprehensive overview of the spatio-temporal representations. They provide various aggregations, like frequency, likelihood, probability, or relative weights. The authors compare the methods in different ways, on different data, and using different metrics, making it difficult to assess their actual performance. To overcome the difficulties in comparison of different approaches to mapping, we follow the idea from our previous study (Vintr et al., 2020). There, we proposed a specific criterion comparing different maps that provided incomparable costs. In this work, we generalize the criterion into the methodology to create different criteria from a chosen cost function that suits the studied attributes of maps. We created an extensive set of generalized approaches to represent the spatio-temporal phenomena covering a large part of the current methods. We applied them to the real-world dataset of 1 month of human detections and compared them using the two criteria following the proposed methodology. The straightforward interpretability of the results provides us with exceptional insight into the strengths and weaknesses of different approaches.

## 2 Related Work

### 2.1 Static Models

#### 2.1.1 Discrete Maps

A straightforward way to include a historical knowledge of human behavior in the robotic map was proposed in the study by (Ravankar et al., 2020). The authors divided the map into the crossings (nodes) and halls (vertices) and projected the map into the graph. A set of cameras scanned every hall, and the visual system detected and counted people in these halls. Each vertex of the graph created a dictionary of observed human movements, which leads to a historical heat map. The field robot experiment proved that navigation using historical knowledge leads to evading congestions. (Nishio and Niitsuma, 2019) included historical information about the frequency of people in every cell and usual directions and speeds and also acceleration in the classical grid-based map. They could detect natural ways people use in larger areas, where the usual paths are not apparent from the hall’s structure. They stated that the knowledge of the acceleration distribution leads to a better understanding of “smooth flows” in a human movement. In the study by (Senanayake and Ramos, 2018) the authors proposed a directional grid map (DGM) that models the distribution of directions and speeds in the occupancy grid. Each cell applies an expectation-maximization algorithm to estimate the mixture of von Misses distributions from historical data.

#### 2.1.2 Spatially Continuous Maps

(O’Callaghan and Ramos, 2012) argued that a continuous spatial map has better properties for robot navigation than a classical grid map. They proposed Gaussian process occupancy maps (GPMOs) that overcome single-scaled maps’ problems and provide accurate maps even with relatively sparse and noisy data. However, such an approach cannot be applied to modeling directions. It suffers from averaging angles, leading to meaningless predictions when humans traverse positions in both directions (McCalman et al., 2013). As a solution, they proposed a combination of the Kernel Bayes’ Rule and the Gaussian mixture pre-image recovery method (KBR-GM). Another approach to overcoming weaknesses of discrete occupancy maps was proposed in the study by (Kucner et al., 2016). The authors did not use Gaussian processes mainly because they generally fail to model multi-modal distributions. Instead, they modeled the continuous distribution of measured directions and speeds in every cell of a grid using an expectation-maximization method based on the Independent von Mises–Gaussian distribution (Roy et al., 2012). Then they defined an interpolation technique that provides a distribution estimation at any position. They called it CLiFF-map and applied that to the people tracking and wind data. In the following work (Kucner et al., 2017), they trained the CLiFF-map with sparse data (75 and 3%) and then reconstructed it to the original resolution using Monte Carlo and Nadaraya Watson methods. They proved that it is possible to gather relatively accurate models from very sparse data. Later, they proposed a robot motion planner over the CLiFF-map based on RRT* (Palmieri et al., 2017). They defined the Extended Upstream Criterion (EUC) as a cost function to favor the robot’s movement with the vector field. The trajectory found in such a way is most likely to offer a good trade-off between length and control effort against the dynamics of the environment. They showed that the model of the movement of people could be used to navigate the robot through the crowd effectively. In the study by (Swaminathan et al., 2018), CLiFF-map was extended with a Down-The-CLiFF (DTC) cost function for trajectory planning, which explicitly accounts for the environment’s dynamics and the uncertainty in the flow model. The cost function incorporates observation and motion ratios, which, compared to the previous EUC, favors the less crowded areas and provides information for an exploration task.

### 2.2 Short-Term Dynamics in Maps

In the aforementioned approaches, historical knowledge does not provide any information about the changes in the dynamics of an environment. One of the approaches is to include the short-term changes in the map derived from the actual observation of the situation. In the study by (Kucner et al., 2013), the authors proposed a Conditional Transition Map (CTMap), which models in every cell the probability distribution of vehicle transitions between the last and the next cell. As the behavior of the usual vehicle is not random, the CTMap estimates the probability of the exit direction of the vehicle from the entry direction. Using the Conditional Probability Propagation Tree (CPPTree) graph representing all reachable transitions, the method can predict the vehicle’s trajectory that entered the scene or provide a convenient navigational plan for a robot. Another discrete short-term model can be found in the study by (Wang et al., 2014), where authors predict the path of a walking person based on his actual position in the environment using the input–output Markov model. The model that estimates a human’s future position directly from observations (Kollmitz et al., 2015) allows for reactive navigation that respects a human’s personal space without the need of repetitive recalculations of the robot’s trajectory. (Senanayake et al., 2020) proposed a static spatial map that includes distributions of directions and speeds of the vehicles at the crossroads. It is used prior to the trajectory prediction, which is updated based on the current observation. The current observation, for example, the blinking of a car, is then used to estimate the new directional distribution and, eventually, the most probable path of the vehicle. (Nardi and Stachniss, 2020) proposed a graph that represents an environment and which edges were traversable or not during a robot’s task. The model learns which edges were traversable together. Based on this model, they can predict where the robot can go after observing a small part of the environment. Then, the information is exploited to provide better navigation plans. With the advancement of the neural networks that include long short-term memory, one can find in the literature a vast amount of the methods for short-term predictions derived from the actual observation (Rudenko et al., 2020).

### 2.3 Partly Discrete Spatio-Temporal Maps

#### 2.3.1 Maps With Discrete Temporal Domain

Apart from modeling the directions of the movement of particles in the environment, the scientific community focuses on modeling the changes in the environment over time. A prevalent approach to modeling a time-dependent phenomenon is to create seasonal windows (usually 1 day long) and to model the environment over the different parts of these windows (Van Laerhoven et al., 2008; Blanke and Schiele, 2009). This approach models patterns of the environment change, but it is necessary to define the principal periodicity, that is, the length and the resolutions of windows. In the study by (Bennetts et al., 2019), the authors analyzed the flow of the wind. They proposed a continuous AirFlow Map and called it stf-AFM. It is a set of spatio-(short)temporal models inserted into the “calendar”. The authors are searching for periodical features over the calendar using a combination of autocorrelation, fast Fourier transform, and clustering over the frequencies. They pointed out that “knowing periodicities could have some impact.” A bachelor thesis (Kubiš, 2020) proposed a method of time-window GMM applied to the taxi demand prediction. Contrary to the previous article, the essential periods were gathered from the data in advance and used for creation of proper time windows. The method then estimates the spatial distribution using EM-GMM in every time window. The author showed that it is possible to predict the demand for a few weeks to the future. A comparable method was also used for ambulance call forecasting (Bayisa et al., 2020). The authors proposed a spatio-temporal log-Gaussian Cox process. They divide the prediction task into multiple subtasks for different temporal and spatial classes (7 days of the week, 4 seasons of the year, and 5 regions). In those 140 subsets, they calculate a continuous one-day-long model derived from the historical data of several years.

#### 2.3.2 Smoothing the Grid

However, the time interval usage suffers from the discontinuity at the borders of the intervals, which is necessary to overcome (Chinellato et al., 2017). The bachelor thesis of (Blaha, 2020) describes the system that was created to help people during the early times of the Covid pandemic. The system predicts how crowded places like shops and pharmacies will be (Laboratory of Chronorobotics, 2019). As the learning data were highly sparse and unevenly measured, the author employed a histogram-based temporal model smoothed by spline at every tested place. Despite the meagre quality data, the system could predict the ideal time for shopping in the next few days. The smoothing of the spatio-temporal models is generally a complex task. For example, the authors (Zhang and Zheng, 2020) applied triangulation over the space of a discrete spatio-temporal model and interpolated the distribution linearly over the sub-spaces so that the distribution is piece-wise linear and continuous.

#### 2.3.3 Continuous Modeling of Time Over the Spatial Grid

A specific approach to spatio-temporal modeling is called Frequency Map Enhancement (FreMEn) (Krajník et al., 2017). In one of the experiments, the authors created a topological map. Each place on the map is defined by a set of visual features detected in the past. Every detection gets an ID, and if detected multiple times, it creates a binary time series (detected during observation or not). Every time series is then decomposed by frequency analysis. Each place at any time is then described by a set of the most probably detectable features. It is also possible to represent each place as an occupancy grid where each cell holds the binary time series that specify the occupancy state. All modeled phenomena are understood as static or periodic, which allows predicting future states of any feature at any time. Therefore, it is also possible to predict the most probable position of static and dynamic obstacles. The authors also define the persistence of the features, which allows the map to include unpredicted obstacles that were actually detected. Moreover, an open-source library integrated with ROS for long-term mobile robot mapping called FROctomap (Krajnik et al., 2014a) is available.

Experiments and applications of FreMEn provide the most complex insight into benefits obtainable from incorporating spatio-temporal models into robotics. FreMEn was applied in the task of mapping (Krajnik et al., 2014b). The experiments showed the ability of the method to model binary states with very high precision. Applying it to the occupancy grid map lowered the prediction error compared to the static approach by 60%. Then, it was applied to the localization task (Krajník et al., 2014b). The experiment was accomplished at eight places that were supposed to be distinguished using two different approaches. The first approach described each place using a “fremenized” occupancy grid, and the second approach modeled the places using image features whose detection probabilities were also fremenized. The experiment proved that incorporating the FreMEn improved the localization ability of the robot. They also stated that the most prominent period was most influential in a long-term experiment as it persists over a more extended time. (Fentanes et al., 2015) proposed a topological map in which there was the traversability of the edges modeled by FreMEn. They implemented the time-indexed Navigation Markov decision process that improved the planning of the navigational tasks in the changing environment. In a robotic search task (Krajník et al., 2015a), the performance of Frequency Map Enhancement–based and Periodic Gaussian Mixture–based modeling were compared. The robot was supposed to search for people and objects in three different environments. Compared to the stationary models, the experiment showed a decrease in the search time of about 25% and a decrease of places of about 33% using both proposed methods. The authors speculate that finding periods using FreMEn and approximating the events with the mixture of Gaussian distributions can lead to a better output. Although the fremenized occupancy grid holds the parameters of the binary time series in every cell, it was successfully applied to a directional grid map over a large area of mall halls (Molina et al., 2018). FreMEn was also subsumed into the *Human-Aware Allocation* proposed for cooperation between multiple robots in a human-populated area (Surma et al., 2021). Its predictions were employed in the multilayer *Map of Dynamics* to ensure the optimal division of tasks with human presence in the environment. Considering the ability of a robot that uses FreMEn to recognise “when” it observed the most informative situation, the novel *information-based Monte-Carlo scheduler* for exploration was proposed (Santos et al., 2016). Eventualy, different exploration strategies (Krajník et al., 2015b; Santos et al., 2017; Molina et al., 2019) and exploration–exploitation dilema (Kulich et al., 2016) were studied. In the study by (Jovan et al., 2016), FreMEn was redefined into the Addition Amplitude Model (AAM). The most significant frequencies were iteratively discovered from learning data and actual reconstruction differences, that is, model errors. The main difference to the original FreMEn resides when the frequency with the highest amplitude in the errors is part of the frequencies that established the reconstruction. In such a case, the amplitudes of those two (identical) frequencies are summed up, and the shift is averaged.

### 2.4 Continuous Spatio-Temporal Maps

The main problem of the approach above is the spatial independence of the neighboring cells. It can be addressed by spatial ordering (Cliff and Ord, 1975), but the spatial ordering has to be predefined *a priori* and cannot be changed during the learning process (Shi and Yeung, 2018). The necessity to model spatial dependencies in the dynamic environment leads to a continuous spatio-temporal representation of the environment. Although the continuous models of the environment changes are computationally demanding, they are beneficial due to their memory efficiency (O’Callaghan and Ramos, 2012; Vintr et al., 2019b) and robustness to the outliers (Zhi et al., 2019).

#### 2.4.1 Maps With Limited Number of Periods

The continuous spatio-temporal models were successfully used in ambulance demand prediction. (Zhou et al., 2015) proposed a time-varying Gaussian mixture model. They created 2-h windows with a 1-week periodicity, and applied GMM in each window. Moreover, they apply constraints for the weights of each Gaussian in a way that timely preceding and subsequent weights, and weights from identical time windows of preceding and subsequent days, directly influence the current weight. In this way, they also include 1-day periodicity and smoothness to the model change in their model, which they refer to as *short-term serial dependence*. Later, they proposed a warped kernel density estimation model that produces geometrically better density estimation than time-varying GMM (Zhou and Matteson, 2016). The warping kernels are derived from spatial geometry. The spatial positions of the ambulance demand constitute clusters projected to a weighted graph as nodes. The edges are derived from the highways and roads, and weights correspond to the usual traffic. The parameters of the graph then influence the shapes of the spatial clusters. Such an approach provides a “fit to structure” distribution. The fundamental periodicity in the proposed model was 1 week. Due to the computational complexity, the model used an 8-week-long sliding window for learning while prediction was tested in the consequent 4 weeks. Continual learning also served as “forgetting” and provided different predictions in different seasons (winter/summer). However, the model did not prove its improvement over initial methods when applied to a crime prediction in Bogota (Garrido Mejía, 2018).

In the following work (Pabón et al., 2020), kernel warping was applied to the homicide prediction in Bogota. The authors explain the inefficiency of the model in several ways: the relatively low amount of homicides, changes of positions of problematic groups across historical data, and the direct impact of patrolling strategy of police known as “runaway feedback loops.” They solve the known issues by enriching the data with street fights, which are very connected to the homicides, and including temporal decay components in the studied models. Temporal decay helped all the models, and the kernel warping model provided the best predictions. In the study by (Zhou and Matteson, 2015), the spatio-temporal ambulance demand is modeled in a way that the dataset is divided into spatio-temporal cells. By applying autocorrelation to the data, the authors estimated two main periods. Then those periods were exploited to create a continuous spatio-temporal model by applying kernel-based GMM to the weighted aggregations in the cells. The weights were derived from the distances of cells in time-considering found periods. The method is referred to as spatio-temporal kernel density estimation (stKDE). stKDE was also used for a modeling ambulance intervention in Milan (Gilardi et al., 2021). The authors pointed out that it is possible to use fast Fourier transformation instead of autocorrelation to estimate periods in the data. In the study by (Nilsang and Yuangyai, 2021), a spatio-temporal model based on stKDE with one periodicity was used as a part of the method for an allocation of ambulance bases.

Spatio-temporal models were also applied to the spread of disease prediction. In the study by (Senanayake et al., 2016), the authors modeled the propagation of influenza. They created a spatio-temporal model based on Gaussian processes consisting of spatial, temporal, and spatio-temporal components. Except for the temporal decay, the model also consisted of the 1-year periodical temporal component. The periodicity was chosen based on general knowledge. Similarly, a continuous spatio-temporal map of disease spread over Turkey consisted of 1-year periodicity (Ak et al., 2018). The authors stated that the continuous spatio-temporal model could predict the disease spread in unmeasured regions inside Turkey in the future.

#### 2.4.2 Periods Gathered From the Data

Although the aforementioned models were continuous and spatio-temporal and included seasonality, only one or two periodicities were employed. In many cases, the periodical component was obtained not from data but *a priori* knowledge or expertise. In long-term robotics, a robot has to obtain the natural periodicities of the dynamic environment from its observations, and some of them are likely out of usual human expertise. Moreover, some human-populated environments do not necessarily follow the day/week/year pattern. The flow of continuous media was modeled in the study by (Guizilini and Ramos, 2015) by applying Gaussian processes to the measured data. The algorithm used a covariance matrix formed by the spatial and temporal components. The periodical temporal components were obtained iteratively using frequency analysis over the model’s errors. The temporal components also include temporal decay. The spatial and temporal components of the covariance function are calculated separately and multiplied at the end. Nevertheless, the computational complexity of the Gaussian processes grows fast with the number of data points (Jovan et al., 2016; Senanayake et al., 2021). The extraction of multiple periodical features from the data was targeted in the study by (Tompkins and Ramos, 2018) proposing Fourier Feature Approximations for Periodic Kernels and its multidimensional variant (Tompkins and Ramos, 2020). Although it was not applied to the spatio-temporal modeling, together with formerly proposed Hilbert maps (Ramos and Ott, 2016) that were subsequently improved to be updated incrementally (Senanayake and Ramos, 2017), it should be taken into consideration.

In the study by (Krajník et al., 2019), the authors proposed a continuous spatio-temporal model based on a projection of linear time into the closed subset of higher dimensional vector space, warped hypertime. The projection has its roots in the seasonal windows, but the windows are coiled into circles. Such an approach ensures continuity of the model on the edges of a window. Projected circles form a multidimensional Clifford hyper-torus (Leonard, 2020). The seasonality is obtained iteratively from an error of the actual model. The error forms a time series that is analyzed using FreMEn. The FreMEn returns the most dominant periodicity found in error, and the periodicity forms a new circle. The authors then showed in experiments that the proposed method is equal to or better than FreMEn when applied to the robotics tasks. In the study by (Vintr et al., 2018), Warped hypertime was applied to the task of detecting anomalies. It was proved that the model learning on binary data is faster and converges to a better quality model than in other state-of-the-art methods. Later, it was applied to the detection of novelties (Rektoris, 2021) in one-dimensional time series without any trend, and it proved the similar quality of the model to the state-of-the-art regression methods while performing better in modeling time series with multimodality. Applied to a human presence prediction (Vintr et al., 2019b), it showed high memory efficiency with a slight boost to the prediction compared to the discrete methods. In the study by (Vintr et al., 2019a), the hypertime version derived from the binary version was applied to the directions of people. To avoid ambiguity caused by averaging angles, the model’s spatial part was four-dimensional—the method also modeled speeds, that is, velocities, and the resulting angles were calculated by integrating velocities over the angular intervals. It was not computationally rational to calibrate the model using a grid of aggregations similar to the previous work (Vintr et al., 2019a). Instead, the authors extended the data with “negative measurements;” they added random noise labeled as “not a human.” Two models (humans and artificial nonhumans) were learned, and the “calibration” was done similarly to the approach used for binary data (Vintr et al., 2018). Although the addition of artificial zeros is meaningful in some two-dimensional spatial cases (O’Callaghan and Ramos, 2012), generally, it is not a convenient approach—weigh how many possibilities of “not detected velocity” one has to take into consideration.

### 2.5 Benchmarking Spatio-Temporal Maps

The authors that propose new mapping methods apply a wide variety of quality measuring techniques. It is not uncommon to provide only a discussion about the visual quality of the map regarding the most common directions (Kucner et al., 2013, 2016), reconstructed signals (Fentanes et al., 2015), or changes of the heat map over time (Vintr et al., 2017; Nilsang and Yuangyai, 2021). Such an approach is usually used to provide insight into the proposed concept’s basic behavior.

A very popular measure of the quality of discrete or discretized maps is mean square error (Senanayake et al., 2016; Yan et al., 2017; Senanayake and Ramos, 2018; Krajník et al., 2019) and rooted mean square error (Guizilini and Ramos, 2015; Jovan et al., 2016; Zhou and Matteson, 2016; Vintr et al., 2019a; Senanayake et al., 2020). Apart from the fact that, in general, the maps not providing the frequency of measurements need to be normalized (Ak et al., 2018), the difference between maps is very small (Vintr et al., 2019a, 2020), which leads to the necessity to enhance the differences of results (Vintr et al., 2019b), and the rank of methods is dependent on the coarseness of the grid (Kubiš, 2020). Similarly, a popular measure is an average (negative) log-likelihood that can be applied to the continuous maps (McCalman et al., 2013; Zhou and Matteson, 2015, 2016; Senanayake and Ramos, 2018; Zhi et al., 2019) but is only meaningful when a probability measure can be derived. It suffers mainly because the map is tested only against detections, that is, in places with testing data points.

Many authors treat their maps as binary predictors and test their quality using measurements derived from the confusion matrix. For the predictors with a predefined threshold, we can find a ratio of true positives (O’Callaghan et al., 2011; Krajnik et al., 2014c), ratio of false positives (Molina et al., 2018), hit rate (Garrido Mejía, 2018; Pabón et al., 2020), and accuracy (Massey, 2019). Some authors do not predefine the thresholds and use measures like an area under the curve (O’Callaghan and Ramos, 2012).

We can find also unique measurements of a map quality like the Pearson correlation coefficient (Ak et al., 2018), Cramer-von-Mises criterion (Bennetts et al., 2019), Kullback–Leibler divergence (Rudenko et al., 2020), and k-NN Universal Divergence Estimator (Kucner et al., 2017). Rarely, we can find average probability density (Senanayake and Ramos, 2018; Senanayake et al., 2020) and chi-square distance (Vintr et al., 2019a; Molina et al., 2019; Stuede and Schappler, 2022).

In the robotic community, there exists also the possibility to compare the maps in simulations (Palmieri et al., 2017; Nardi and Stachniss, 2020), or simulated robotic tasks built on the real data (Krajník et al., 2014c; Krajník et al., 2015a; Krajník et al., 2017; Vintr et al., 2020). The ultimately self-evident measures of the quality of the map then come with real robot experiments (Krajník et al., 2017; Ravankar et al., 2020).

## 3 Generalized Natural Criterion

### 3.1 Genesis of Criterion

The question of the proper evaluation of spatio-temporal models for service robotics has been highlighted in previous work (Vintr et al., 2019a; Vintr et al., 2020). Some authors strongly imply that generic criteria used to quantify the success of regression methods, for example, mean squared error (MSE), are unsuitable for this task (Wang and Bovik, 2009; Vintr et al., 2019a; Kubiš, 2020). It is mentioned that these criteria struggle to differentiate between state-of-the-art methods meaningfully and are not reliable. Small changes in the hyperparameters of the testing procedures can cause significantly different results, even changing the rank of the methods. More importantly, they do not provide a useful proxy of the measure of how well the people in a robot’s environment perceive the robot’s behavior, that is, some *robot acceptance* cost (RA), as they generally have no connection to the application (Wang and Bovik, 2009).

In this work, we build upon a criterion called *expected encounters* (EE) developed in the study by (Vintr et al., 2020). First, we define it according to (Vintr et al., 2020), and then we generalize the ideas to get a universal framework for defining similar metrics for evaluating various robotic maps. Then, we define in detail two specific criteria, which we use in this study to compare methods.

The metric falls into the class of utility metrics, and its primary goal is to measure the usefulness of the learned model for the robot’s navigation in human-populated environments. The EE criterion specifically tests the method’s ability to model the phenomena of human presence in time and space. It does so by presenting a robot with a trained model and a set of navigational tasks that happen at different times. The robot is then supposed to plan its path through the environment for all these tasks minimizing the number of people it expects would cross its path. Path planning tests the method’s ability to model the phenomena in the spatial domain. Another principal idea is introduced to test the temporal domain: the *servicing ratio*. The robot is asked to select a specific percentage of times out of all the times presented where it will execute the navigational task, again to minimize the expected number of encounters with people. All the selected plans are then executed and compared with the positions and movement of people in the testing dataset. The testing environment calculates the encounters between the ‘blind’ robot and ‘blind’ people—the system does not provide any artificial reaction of any element involved.

To formally define the original EE criterion, we first define the *service disturbance function* SD(*r*) with the parameter *r* ∈ [0, 1], which we interpret as the *servicing ratio*, that is, the ratio of navigational tasks the robot is required to perform. The robot is given a set of navigational tasks at times ${\left\{t_{i}\right\}}_{i=1}^{N}$ , and for each one of them, it plans a path with cost *c*_*i*_ defined by the spatio-temporal model of human presence to be evaluated. The testing dataset then, for a given trajectory, gives the number of weighted encounters *e*_*i*_ (see Section 4.2.1) that would occur for the given planned trajectory. The *service disturbance function* is then given as follows:

$$SD\left(r\right)=\overset{\left\lfloor rN\right\rfloor }{\underset{k=1}{\sum }}e_{\pi \left(k\right)},$$ (1)

where *π* is a permutation which orders times *t*_*i*_ so that *∀k*: *c*_*π*(*k*)_ ≤ *c*_*π*(*k*+1)_, where *c*_*i*_ corresponds to *t*_*i*_, as defined above.

The interpretability of the *service disturbance function* alone provides us with quite an interesting insight into the performance of individual models. To arrive at a single-value aggregation of this function, we adopt the interpretation where we understand this function to be the quantile function of the ordered human–robot encounters. The quantile function allows us to compute the expected value of the distribution, which we denote *expected encounters*, and we compute it as follows:

$$EE=\int_{0}^{1}SD\left(r\right)dr.$$ (2)

Note that the service disturbance function satisfies the necessary conditions of being cumulative and defined on the [0, 1] interval.

### 3.2 Generalized Definition

The criterion was explicitly developed for comparing different approaches to the spatio-temporal modeling of pedestrian flows (Vintr et al., 2020; 2019a) with the necessity of evaluating the predictions producing very different values. Consider the outputs of histograms over seasonal windows (Blanke and Schiele, 2009), CLiFF-map using different cost functions (Palmieri et al., 2017; Swaminathan et al., 2018), Time-window GMM (Kubiš, 2020) producing the values limited only from below by zero, binary-map approaches based on Frequency Map Enhancement (Krajník et al., 2017), and probabilistic STeF-map (Molina et al., 2019). However, the original criterion focuses only on one specific aspect of human-aware navigation. There are a lot of different opinions as to what criterion influences the acceptability of a robot in human society most (Talebpour et al., 2015; Kostavelis et al., 2017). Besides, the acceptability need not necessarily be the only point of view that defines the quality of performing the task (Krajník et al., 2015a; Fentanes et al., 2015; Kulich et al., 2016).

The general idea of comparing the spatio-temporal maps is applicable and helpful in comparing different robotic models predicting diverse phenomena useful in miscellaneous robotic tasks. There are two essential prerequisites needed for the application of the idea of the criterion—a predictor producing (at least) ordinal values that are tailored for the robot to decide on its task, and a measure of the impact of the robot’s decision usually denoted as a *cost*.

To formalize the generalized definition, we start by having a specific task for the robot to perform, during which it relies on information provided by a model and an observable measure of how expensive it was for the robot to perform its task—the *true cost*. This true cost can but does not have to be directly tied to the predictions of the model—a *predicted cost*. This task is supposed to be performed under different values of the model’s explanatory variable (e.g., time or space) ${\left\{v_{i}\right\}}_{i=1}^{N}$ and for every *i*, the robot acquires a predicted cost *c* (*v*_*i*_) of its hypothetical task from the predictor. Then, we define a *true cost function* C:{1,…,N}→R , which provides a true cost for every *i*. Finally, we define a *quantile function* of the distribution of the ordered true costs as follows:

$$Q\left(r\right)=\overset{\left\lfloor rN\right\rfloor }{\underset{k=1}{\sum }}C\left(\pi \left(k\right)\right),$$ (3)

where *π* is a permutation, which orders indexes *i* so that *∀k*: *c* (*v*_*π*(*k*)_) ≤ *c* (*v*_*π*(*k*+1)_) All interpretations like the *servicing ratio* hold in the generalized case as well and it is again possible to compute the *expected value* of the ordered true costs:

$$EC=\int_{0}^{1}Q\left(r\right)dr.$$ (4)

### 3.3 Methodology

We strongly disagree with applying statistical tools developed to describe or compare repeatable experiments to experiments involving people. The same applies to other uncontrollable entities, like animals, wind, or traffic. For human-aware robotics, we need to follow the general idea of natural experiments (Dunning, 2012) and apply the methods in real-world situations. However, autonomous robotics is not in the development stage in which the field robot experiments can be accomplished securely in large enough repetitions to provide comparable results. We suggest that the comparison should be performed (at least) in the simulations built on the real-world data. In these simulations, we can repeat the same real situation and apply the different methods.

The proposed generalized definition of the criterion can be applied to various robotic tasks a robot performs. We need to specify the predicted costs *c* (*v*_*i*_) and the true cost function C that provides the true cost during the robot employment in the simulated, real-data–based experiment. Using the proper definition of the true cost function, we can compare the approaches and specify which aspect of the proposed approach is better than another. Moreover, we can also elaborate on what aspects should be compared by designing new criteria following the generalized one. As the proposed criterion and its usage are derived from the general idea of natural experiments, we denote it the *Generalised Natural Criterion*, *GNC*.

The general template for applying GNC for a particular predictor then has the following steps:

1) execute or simulate the experiment with different settings of the investigated environment,
2) order the measured costs by the predictions of the method and accumulate it, as described in Eq. 3, and
3) calculate the expected value of the cost of the robot’s behaviour, as in Eq. 4.

### 3.4 Application of GNC

GNC and the concept described above are generally applicable to the scenarios where the robot can estimate what the cost of solving the task in different situations is. The predictor providing the estimation uses some explanatory variables as input, like a relative position of a robot to an obstacle, the time of task execution, or actual weather.

Then, we need to define a function, referred to as true cost function C , which provides a measurement of the quality of the performed task. For example, in the study by (Kostavelis et al., 2017), the quality of human-aware navigation is measured by the mean distance between the moving human and the robot, the time spent in areas associated with the personal zone, the human discomfort, and the total navigation time needed for the robot to reach the desired goal. Such true costs are directly applicable, and the different approaches to solving the problem can be compared. In the case of testing different spatio-temporal maps (Vintr et al., 2020), the authors defined the true cost function as the cost of the optimal path.

We mentioned that the predictor needs to produce at least ordinal values. The optimal predictor produces real values with the probability of obtaining identical values close to zero. The following sections discuss some scenarios where the direct application of the methodology defined above is not straightforward because the predictions are not real values. It needs some additional effort to utilize the method entirely.

#### 3.4.1 Binary States

Many environmental models in mobile robotics are composed of independent binary states, such as the presence or absence of people, the visibility of landmarks, and the traversability of certain areas (Krajník et al., 2017). As an example, let us consider the task of traversing an environment using its time-varying topological map as quickly as possible (Fentanes et al., 2015; Nardi and Stachniss, 2020). Although the robotic methods usually provide more nuanced prediction than just 0 or 1, for example, a probability of the state being 1, it happens that a confident predictor forecasts that the traversability in more than one situation will be 0 or 1. In such a case, the ordering needed in Eq. 1 would have several different solutions. We can divide the predictions into successful (1) and unsuccessful (0) classes and understand the values of 0 and 1 as an ordinal variable. Inside those classes, the ordering can be done randomly, and the expected cost value can be calculated as an average of multiple random reorderings.

#### 3.4.2 Ordinal Variables

When the target variable is ordinal, the ordering can be solved similar to the binary states by multiple random reordering. If available, the predicted values can also be weighted by the system’s confidence or the probability of each class. Another approach consists of applying a regressor function to predict the targeted value, as in the bachelor thesis by (Rektoris, 2021). Both approaches exploiting the confidence provide a continuous output of the prediction, and therefore, it is possible to order the costs with ease.

## 4 Evaluation

### 4.1 Human Disturbance Experiment

The assessment of how the occurring people in the environment accept the robot’s presence is not straightforward. In some robotic studies, we can see, for example, an effort of the scientists to learn a robot to evade accessing a human’s personal space (Kostavelis et al., 2017) or breaking pair-wise social relations (Okal and Arras, 2016). However, the survey on the experience of people with the long-term autonomous robot (Hebesberger et al., 2017) shows that a major disappointment is caused by robots’ inability to adapt their behavior to the general pattern of human activity. The lack of adaptation results in having to break ongoing social interactions because of a more critical task (e.g., battery charging) or awkward evasion manoeuvres during human–robot encounters in crowded areas. From our observation, the inability of the robot to adapt its activity to the temporal patterns of its workplace causes it to be perceived as unintelligent, awkward, annoying, and useless. Eventually, as a robot provides a service nobody wants at the right time, people start to push it to stay away, turn it off, or otherwise treat it as an unwanted entity.

While a robot certainly needs to adequately react to the arising situations and avoid people in a socially acceptable way, people perceive the robot better if it can schedule and plan its activities to avoid socially inappropriate situations completely.

Our primary hypothesis states that the robot that can decide when to provide a service disturbs people less than a robot without such an ability. To prove the hypothesis, we run a robot in a human-populated environment using two types of navigation systems. The first one used a traditional occupancy grid, containing static obstacles, to plan the paths, which were traversed using an industrial-grade reactive navigation system, provided by the manufacturer of the HSR robot used for the experiment; we call this *Reactive* navigation. The second one, named *Anticipative* navigation, also used the build-in navigation of the HSR robot. However, the paths and times of navigation were planned using a spatio-temporal-directional map of pedestrian flows.

We measured people’s discomfort during the experiment by counting only the strong negative reactions toward the robot, which eventually led to an intentional search for a way to complain. A human that goes and complains was, in our interpretation, so distracted that he changed his original plan and started to solve the unexpected situation—similarly to the strong negative reactions that led to pushing a robot out of society (as mentioned earlier).

#### 4.1.1 Robotic Platform

**FIGURE 1:**
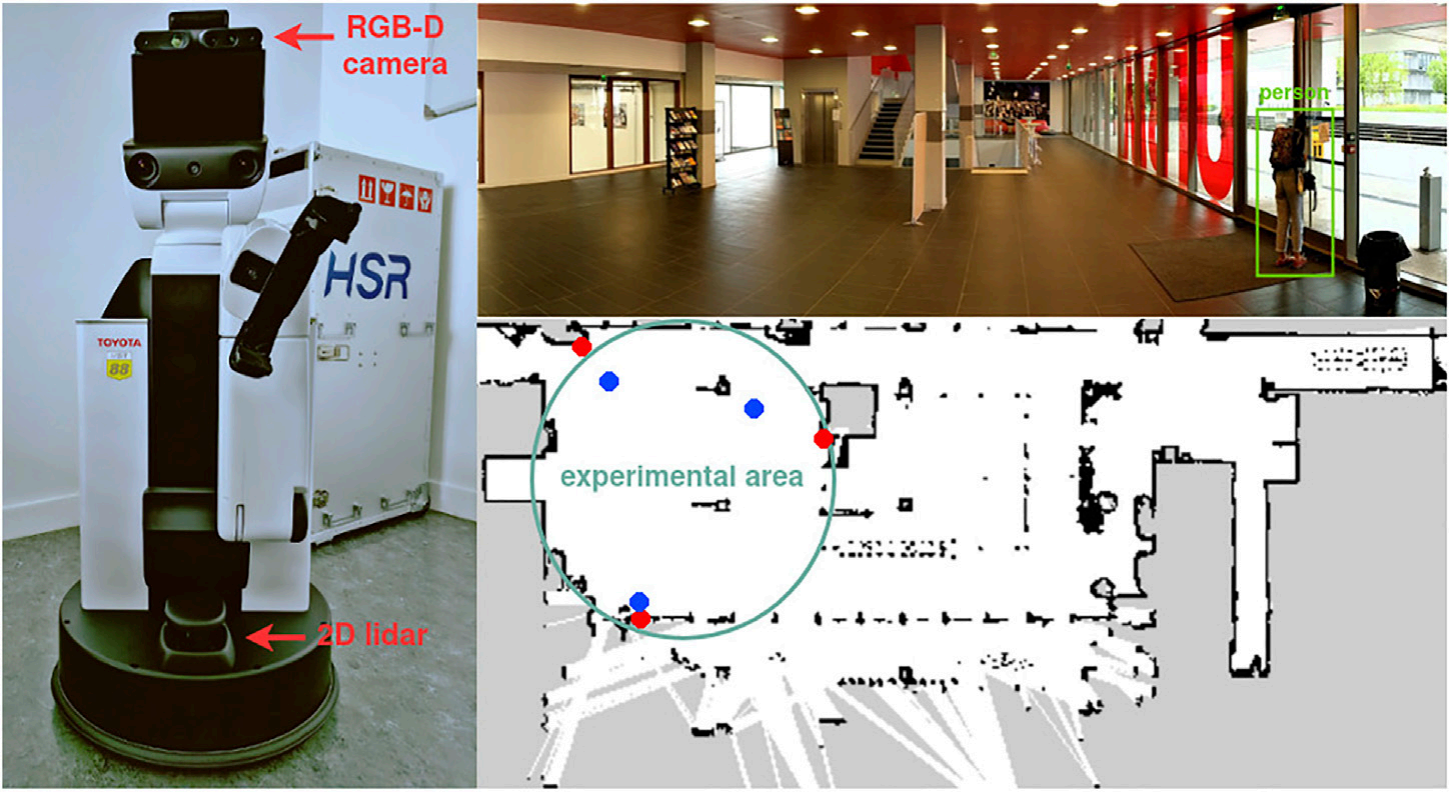
Left: Toyota HSR robot including an Xtion RGB-D camera and a Hokuyo UST-20LX 2D LiDAR. Upper right: experimental environment. Lower right: occupancy grid map for robot navigation. The red points are representative of the positions of the removable tags, and the blue points are the locations of the waypoints.

As shown in Figure 1, a Toyota HSR robot (Yamamoto et al., 2019) was employed for experiments, which is equipped with an Xtion RGB-D camera (for obstacle avoidance only), a Hokuyo UST-20LX 2D LiDAR (for obstacle avoidance and global path planning), and other sensors that were not used in the experiment. The human-aware navigation system was deployed on a laptop with an Intel i7-7700HQ processor and 32GB RAM, wired to the robot, and carried on its back. All the software was implemented into the Robot Operating System (ROS) (Quigley et al., 2009) with high modularity using C++ and *Python* mixed coding, running in real time on Linux Ubuntu 18.04 LTS (64-bit) and ROS Melodic.

#### 4.1.2 Experimental Setup

The real-world experiments were conducted with the HSR robot on the afternoon of the 12th of December and the morning of the 13th of December 2019 and both in the same place, the hall of the UTBM building. The experiments were designed by researchers who do not work at UTBM, and there was no advance notification to anyone involved in the experiment. In each experiment, we allocated two 40-min slots to perform 10 patrols, during which HSR had to visit three predefined waypoints. By integrating our spatio-temporal model with the HSR’s own navigation system, the robot polled three waypoints in a counterclockwise sequence while adapting its movement to the moving people using HSR’s built-in collision avoidance. If people occupy a waypoint, the robot waits until the waypoint is free.

**FIGURE 2:**
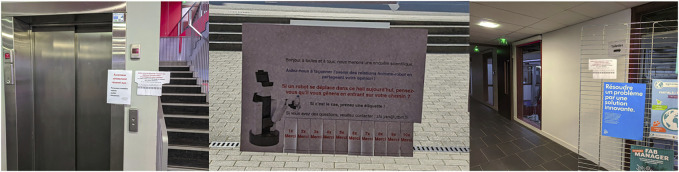
Three paper sheets placed with removable tags near the three waypoints.

Moreover, as shown in Figure 2, three paper sheets were placed with removable tags near the three waypoints. These sheets asked people to remove a tag if they felt that the robot was causing a nuisance by forcing them to avoid it. The idea was to count how many people were distracted by the robot that they performed an intentional operation due to the stressful situation.

#### 4.1.3 Results of Human Disturbance Experiment

**FIGURE 3:**
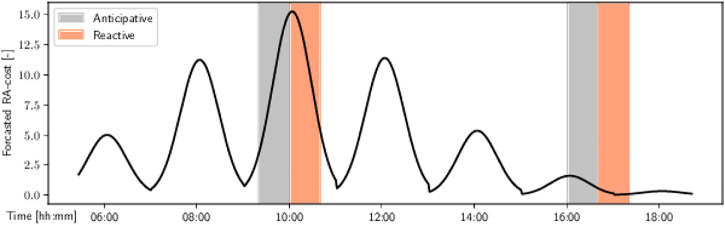
Forecasted (8 months horizon) robot acceptance (RA) cost and timeslots allocated for the experiment. Note that the bottom of the troughs is sharp due to the model not using sinusoids. Courtesy of (Vintr et al., 2020).

The robot was supposed to drive through the environment 10 times in every time slot. Every run took approximately 2 min, which means that the task took up 50% of the assigned time. The reactive navigation went through the hall in uniformly distributed times, covering whole timeslots. The anticipative navigation decided on the proper times by exploiting the predictions from the spatio-temporal map of pedestrian flows (similar to FreMEn_WHyTeS_Clusters, Section 4.2.3). In Figure 3, the graph represents the prediction of the *robot acceptance* (RA) cost in the area, that is, the disturbance to the people caused by hypothetical execution of the patrolling task regarding the prediction of pedestrian flows (Vintr et al., 2020). A *control* experiment was also performed to evaluate how often people would indicate they were annoyed with the robot regardless of its behavior. The robot was removed from the vicinity of the experiment, but the recording apparatus was left in place.

The experimental results are summarized in Table 1. The column ‘People total’ describes the number of people passing through the area within the appropriate time window. The number of people in the hall while the robot was performing its task is listed in the ‘People involved’ column. The last column summarizes a number of paper tags removed by people who considered the robot’s behavior annoying.

**TABLE 1:** Comparison of reactive and anticipative navigation.

Evaluated	Time	People	People	People
**Behavior**		**Total**	**Involved**	**Annoyed**
Anticipative	9:20–10:00	115	17	0
Reactive	10:00–10:40	132	46	2
Anticipative	16:00–16:40	43	6	0
Reactive	16:40–17:20	23	14	1
Control	9:20–10:40	211	-	0

The morning timeslots chosen for the experiment included more people walking through the hall than evening timeslots. The observed distribution of people was not uniform. For example, when the robot was performing its tasks, we manually counted that in the time (sub)slots 9:50–10:00, 10:00–10:15, and 16:00–16:10, there were approximately 70, 90, and 20 people passing through, respectively. It corresponds to the prediction of the RA cost, as shown in Figure 3.

While performing the control experiment, 211 people passed through the hall, and none of them recorded that the robot was annoying them. That indicates that the people passing through the hall were not distracted by the equipment so that anybody would search for a way to complain. The robot taking advantage of anticipative navigation decided to perform all the tasks during time slots 9:20–9:40 and 16:20–16:40. Incorporating the spatio-temporal model into the robot’s planning led to evading the most crowded time slots, as seen in the column ‘People involved.’ The robot incorporating only reactive navigation and performing its tasks uniformly drove through the hall even in very crowded situations when people were in a hurry. The robot’s presence inevitably affected the flow. People were distracted by replanning their trajectories, which resulted in the intentional change of their original plan from ‘to pass the hall’ to ‘to complain,’ as documented in the ‘People annoyed’ column.

We proved that the navigation system that follows human routines distracts people less than the one lacking this capability. We also showed that predictions from the spatio-temporal maps of pedestrian flows provide good enough information to evade socially inappropriate behavior and strongly support reactive navigation. The difference between the anticipative and reactive navigation was so substantial that we did not need to use subtle quantitative metrics developed for the assessment of human-aware navigation methods (Okal and Arras, 2016; Kostavelis et al., 2017).

### 4.2 Comparison of Spatio-Temporal Maps

#### 4.2.1 Two Criteria for Spatio-Temporal Map Comparison

We claim that human-aware navigation needs to include spatio-temporal maps and use them for the decisions in advance of the navigational task. Therefore, we want to compare the maps in their ability to support navigation systems.

The predictions produced by approaches in our comparison (Section 4.2.3) are real values. They are interpreted as the likelihood of human presence at some position in space and time in spatio-temporal scenarios and as the likelihood of people moving with a specific velocity at some position and time in spatio-temporal-directional scenarios. We created simulated scenarios at different times, where the goal for the robot was to visit 3 distinct places in a university hall. The predictions filled a spatial grid with the predictions, and Dijkstra’s algorithm found the cheapest path considering the predictions. We let the system choose the order of the visits in each run, which lets the robot follow the predicted pedestrian flow.

Following our previous work (Vintr et al., 2020), we compare the quality of the spatio-temporal map primarily by the expected encounters *EE* with the associated quantile function SD(*r*), referred to as service disturbance. The *encounters* are obtained using simulated movement of the robot. The robot follows the path obtained from Dijkstra and continually recalculates people in its predefined vicinity every 10 cm. As we hypothesize that the disruption of people is highly correlated with the unexpected replanning of their movement, the contacts with people are weighted *w* by a mutual robot and human direction of movement and referred to as encounters:

$$w=1+\frac{v_{h}\cdot v_{r}}{\left\|v_{h}\|||v_{r}\right\|},$$ (5)

where *v*_*h*_ and *v*_*r*_ are the velocities of a human and a robot, respectively. The weight *w* follows the idea of the upstream criterion (Ko et al., 2014), providing us with information about whether the given model correctly predicts the directions of pedestrian flows. The encounters represent the true costs, while the aforementioned method to obtain them represents the true cost function.

During performing the comparison, we encounter the need for the differentiation between models that force the robot to *always* go around the walls and the models that additionally provide information about when it is possible to choose a short path directly through the hall. Therefore, we defined a second true cost function that provided us with the distance traveled by the robot in each run. We denote the secondary measure of the quality as an *expected length* *EL* and associated quantile function as a *traveled distance* *TD*(*r*). It provides information on the ratio of the chosen paths predicted as ‘safe’ regarding meeting people and the ratio of safe and short paths. Such information can be exploited to estimate the robot’s ability to decide on safe and fast traversals of the environment when using a given model.

In general, the patio-temporal maps provide predictions to the navigation systems, while the reactive navigation solves the current situation a robot encounters. As such, the criteria we used in our comparison are not focused on testing specific manoeuvres like in pure human-aware navigation studies, (Okal and Arras, 2016; Kostavelis et al., 2017) but cover the similar principles directly connected to the specialized human-aware manoeuvres. If the scientist focusing on human-aware navigation recognizes the need for another, more specialized criterion to compare the predictive ability of maps, they can follow the methodology proposed in Section 3.3.

#### 4.2.2 Dataset Collection

**FIGURE 4:**
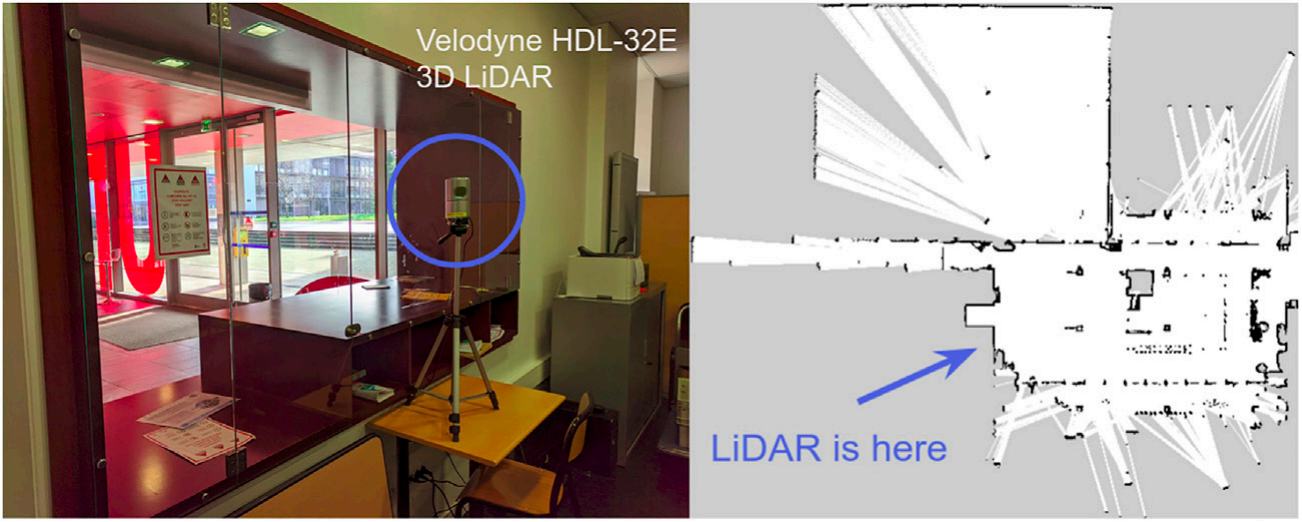
Left: a Velodyne HDL-32E 3D LiDAR placed in the reception near the UTBM building door. Right: the occupancy map built by a Toyota HSR robot (Yamamoto et al., 2019) with a Hokuyo UST-20LX 2D LiDAR.

The dataset collection was performed in a hall of approximately 500*m* ^2^ on the UTBM university campus. As shown in Figure 4, a Velodyne HDL-32E 3D LiDAR was placed in the reception near the building door to ensure safe 24-h operation. The spatial placement of the LiDAR was carefully determined to ensure maximum field-of-view (i.e., approximately 200*m* ^2^) of the hall beyond the glass windows. The raw data of the LiDAR was recorded to ROS *rosbags* 24 h a day.

Our dataset consists of 1 month, March 2019, of continuous human detection used as the training set. The testing dataset consisted of 7 days from 1 week in December. More than 6 million human detections were generated from the LiDAR recordings. They were extracted using the FLOBOT human detection and tracking system Yan et al. (2017). The false-positive detections were removed by searching for extraordinarily stable and immovable human detections. Later, we filtered out reflections of people by manual localization of places producing those reflections. Compared to the dataset used in our previous work (Vintr et al., 2020), the training dataset was twice as long, and the testing dataset did not immediately follow the training period. We also utilized our experience in filtering out the false-positive detections, which led to more effective scripts.

Due to the temporal and spatial continuity of data, the dataset reflects people’s regular activities, like students entering the building lobby at the beginning of class, leaving after class, and eating and chatting in the lobby during lunch break.

#### 4.2.3 Approaches in Comparison

Our comparison focuses on the general principles rather than the optimization of the parameters of the methods tailored directly to the analyzed data. The methods found in the literature can be divided into a few overlapping groups of approaches. Compared to the related work section, we excluded a group of methods incorporating short-term dynamics and included a time series forecasting approach that estimates the number of people over the whole map at a specific time:

1) spatial-only models (Section 2.1) that do not take time into account (Kucner et al., 2016; Senanayake and Ramos, 2018),
2) time series forecasting methods that do not take the structure of the space into account (Vintr et al., 2018),
3) partially discrete and partially continuous models, (Section 2.3.3), which incorporate continuous models in the cells of a predefined grid (Krajnik et al., 2014a; Molina et al., 2018).
4) methods that model spatial and temporal features separately (Section 2.3.1) and understand them as independent (Bennetts et al., 2019; Kubiš, 2020),
5) continuous spatio-temporal methods (Section 2.4.2) that model the spatio-temporal phenomena together (Vintr et al., 2019b; Krajník et al., 2019), and
6) continuous spatio-temporal methods (Section 2.4.1) that model the temporal evolution of the continuous spatial model (Zhou et al., 2015; Zhou and Matteson, 2016).

We implemented various predictive spatio-temporal maps deduced from state-of-the-art principles. The names of the compared approaches are derived from the names of the original methods to make the following text as readable as possible. Our aim was not to systematize the names of the approaches but to maximize the clarity and minimize the time a reader needs to spend learning the differences between approaches’ internals. The description of the methods’ functioning is simplified, and readers who would like to implement them should follow the original proposals. Although some of the compared approaches were not published, they follow known principles, and they are not presented as newly proposed methods.

##### 4.2.3.1 Spatial Models

As a representative of the discrete spatial models, we choose a spatial grid including the ratio of detected people within each cell to all detections, denoted as *MeanGrid*. We also included the *OccupancyGrid* model as a fundamental robotics domain way to represent a map. The occupancy grid captures the environment structure only and neglects dynamic obstacles. Such an approach results in cells with values close to 0 regardless of the number of people moving through them. The continuous spatial model is represented by an expectation–maximization algorithm for fitting a mixture of Gaussian models (Dempster et al., 1977) with its probabilistic prediction, referred to as *GMM*.

##### 4.2.3.2 Time Series Forecasting

We included 3 time series forecasting methods recently applied to forecasting human presence and pedestrian flows. Those methods can be viewed as spatio-temporal models, whose spatial map is *OccupancyGrid*. Frequency Map Enhancement *FreMEn* (Krajník et al., 2017) is derived from the Fourier transform as it was initially proposed for binary data containing binary states. It had to be reimplemented for data that do not contain states but events. It should be noted that although the computational effectiveness of our implementation was greatly enhanced, the predictive ability was lowered due to the inaccuracy of the estimation of heights of amplitudes. (Krajník et al., 2019) proposed a method for time series forecasting referred to as warped hypertime. Based on the definition, we incorporated its two variants, hypertime *HyT* and warped hypertime *WHyTe*. Those methods exploit the ability of *FreMEn* to detect prominent periods in data. They project time into the multidimensional vector space forming a Clifford torus and apply *GMM* with one component to the projected data. The most prominent periods are gathered iteratively by applying *FreMEn* to the series of errors between reconstruction and the measurements. They differ in a metric used for the distance calculation. While *Hyt* uses Euclidean metric, *WHyTe* uses cosine metric. We also wanted to include Prophet (Taylor and Letham, 2018) as a representative of a time series forecasting method that not only can analyze time series with an unequal step between measurements but is also able to model trends. However, we faced difficulties applying Prophet on such extensive data with predictions too far into the future, and we subsequently removed it from the list of the compared methods. The only discrete forecasting method used in the comparison was histogram over the week-lengthy time window with 168 bins, each covering an hour, *HistWeek*.

##### 4.2.3.3 Continuous Models in the Grid Cells

Every aforementioned forecasting method can be employed in the spatial grid forming spatio-temporal models similar to the original idea of the ‘map enhancement’ in the study by (Krajnik et al., 2014b). We included *FreMEnGrid* in the comparison as a representative of continuous ones in the temporal domain and *HistWeekGrid* as a fully discrete spatio-temporal model. We omitted *HyTGrid* and *WHyTeGrid* as their computational demand in the stage of prediction was considerably higher than that of the previous two, and the straightforward optimization of code would violate the design of the testing environment. The third not fully continuous model included is *time_window_GMM* (Kubiš, 2020) which expands *HistWeek* with *GMM* connected to each cell. Its prediction then consists of *GMM* predictions in each cell weighted by *HistWeek* forecast.

##### 4.2.3.4 Independent Modeling of Spatial and Temporal Features

Modeling space independently to time can sound illogical. On the other hand, considering robotic topological maps, we can assume that different nodes will show standardized behaviour that is sometimes more relevant and sometimes almost unnoticeable. We included 7 combinations of spatial and temporal models, whose predictions are multiplications of their independently modeled components: *HistWeek_X_GMM* with a discrete temporal and continuous spatial model, three models including a continuous model of time and a discrete model of space, *FreMEn_X_MeanGrid*, *HyT_X_MeanGrid*, and *WHyTe_X_MeanGrid*, and three continuous maps, *FreMEn_X_GMM*, *HyT_X_GMM*, and *WHyTe_X_GMM*.

##### 4.2.3.5 Continuous Spatio-Temporal Models

The continuous maps that model the time and space together show significant differences in the computational demand. The most demanding are spatio-temporal models based on hypertime. Although they proved their ability to model space-time effectively (Vintr et al., 2019b; a; 2020) by means of the model quality and size of the model, their use on real robots is questionable since their iterative nature consumes many recourses. We included *FreMEn_HyTS_clusters* derived from the *HyT* algorithm and *FreMEn_WHyTeS_clusters* derived from the *WHyTe* algorithm. Contrary to their time series forecasting variants *HyT* and *WHyTe* that iteratively choose the best periodicity, our implementation of those spatio-temporal variants first applies *FreMEn* to the data to gather the most prominent periods and then project data to the hypertime-space. Both apply *GMM* over the projected data to get the model regarding respective metrics. We also included ‘lightweight’ versions that apply *GMM* using only one component, and we denoted them as *FreMEn_HyTS* and *FreMEn_WHyTeS*, respectively.

##### 4.2.3.6 Temporal Evolution of Continuous Spatial Model

We also included continuous spatio-temporal approaches that first fit the structure of the space and then model the temporal changes, similar to the work of (Zhou and Matteson, 2016). *WHyTened_kMeans* divides the space into disjunct subsets using k-means clustering algorithm and applies *FreMEn_WHyTeS* to every part. The second method, *HyTted_GMM*, apply *GMM* to the space, and its every component defines the time series by the measurements matched with it. Those time series are then modeled with *HyT*. The predictions in both methods are calculated as a multiplication of the clustering algorithm prediction and the spatio-temporal or temporal model prediction. The *WHyTened_kMeans* can be understood as multiple disjunct spatio-temporal models, while *HyTted_GMM* represent continuous spatio-temporal models where each component has its specific temporal characteristics.

#### 4.2.4 Testing Environment Setup

The experiments were conducted in a general testing framework with a unified interface available for individual methods. The framework consisted of four stages: training, prediction, pathfinder, and simulation.

The predictors were trained over spatio-temporal and spatio-temporal-directional data during the training stage. There are approximately six million detections of people in the training data. We also included scenarios where the training dataset includes only one per mile randomly chosen detections to provide insight into how robust different methods are to data sparsity.

During the prediction stage, the framework created a spatio-temporal grid over the testing week. The week was broken into 40-s windows. For each of these windows, the area of approximately 200 m^2^ was broken into a grid of 0.25-m^2^ cells. Each spatial cell consisted of 8 directional cells. The predictors filled the directional cells with their estimations of how likely a human would be to go at that time and place in that direction.

The pathfinder applies the Dijkstra search algorithm for each time window, where the cost of a transition is determined by prediction from the tested method. The cost for Dijkstra at a specific position is calculated as an estimation of the cost of an encounter multiplied by the distance to the next state. The paths chosen in each time window are saved together with their lengths and costs. Note that the overall length of paths planned during the whole testing week was hundreds of kilometres.

The last stage of the evaluation is a self-written kinematic simulator. The simulation is done per time window for each model while saving the intermediate results for correctness verification and recording the runtime duration. The robot moves by the chosen path at a predefined speed. The simulator calculates weighted encounters with human detections on the robot’s trajectory. The sum of weighted encounters is saved together with length and path cost, which allows for criteria application.

The framework allows a parametrization of the robot’s movement and goal placement for planning. The experiments were evaluated with the following settings: the robot radius is 1 m, which covers the hypothetical robot radius together with a human radius, and the robot speed is 0.5 *ms* ^−1^.

Some compared approaches also required parameter setting. The adjustment of the parameters was not part of our investigation. The number of components for algorithms applying *GMM* (and k-means) was set to 10 except for *FreMEn_HyTS_clusters* and *FreMEn_WHyTeS_clusters* where we set 5 components (to shorten the computational time). The number of periodical components was set to 5 in every method employing *FreMEn*. We used a computational (floating point) precision of 64 bits to compare and order continuous values.

#### 4.2.5 Results of Comparison

**FIGURE 5:**
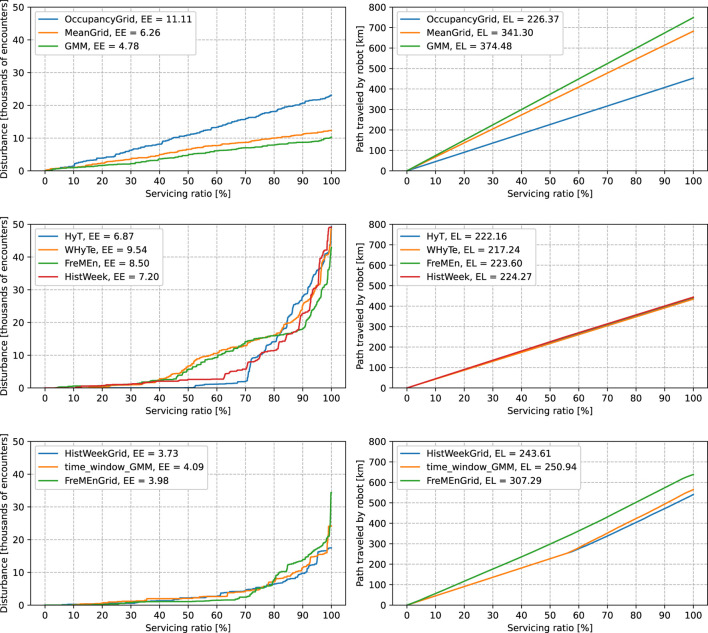
Comparison of different models in the spatio-temporal scenario. The left columns of graphs depict service disturbances, the quantile functions of the ordered human–robot encounters. The right columns of graphs depict traveled distances, the quantile functions of lengths of paths. The labels in graphs consist of names of models and the expected values.

The comparison of methods applied to spatio-temporal data can be seen in the subgraphs in Figure 5. The predictors predicted how likely the robot was to meet a person at a given space and time coordinates. In each row, we have two subfigures, with the left one depicting a graph of the dependence of the service disturbance on the servicing ratio and the right one the length of the path on the servicing ratio.

In the top row of Figure 5, we compare the quality of the predictions of the spatial-only models. None of them considers time, which leads to a random ordering of the costs of the paths. Therefore, the dependence of the service disturbance on the servicing ratio grows linearly. Similarly, as the space models do not change in time, the traveled distances also grow linearly. The *OccupancyGrid* predicts only zeros, and therefore, its expected length of paths is the shortest possible out of all models. *GMM* forces the robot to go closer to the walls than *MeanGrid*, which leads to longer paths and lower expected encounters.

In the second-from-top row, we compare models that take only time into account, which means that those models can order the costs of the paths, but they always use the shortest path possible. Such predictions lead to linear growth of the traveled distance identical to the *OccupancyGrid* but non-linear growth of the service disturbance. All methods can estimate the safest 10% of the time windows to provide the service, while the most successful, *HyT*, can predict 50% of the safest time windows. *WHyTe* and *FreMEn* perform similarly and *HistWeek* is closer in performance to the *HyT* than to the other two models. The rank of the quality of prediction of the compared methods changes at 70% of the servicing ratio. At a servicing ratio of over 90%, *FreMEn* performs best.

In the third row, we compare spatio-temporal models that are fully or partially discrete. *HistWeekGrid* performs similar to *time_window_GMM*. Both models force the robot to follow the shortest paths until a servicing ratio of almost 60%. Then, their predictions start to force robots to follow a longer path, evading the most critical parts of the hall. Similar to the spatio-only models *GMM* and *MeanGrid*, *time_window_GMM* forces the robot to travel longer paths than *HistWeekGrid*. The *FreMEnGrid* model is better than the other two models in scheduling until more than 70% of the servicing ratio, but at higher servicing ratios, it loses its edge.

**FIGURE 6:**
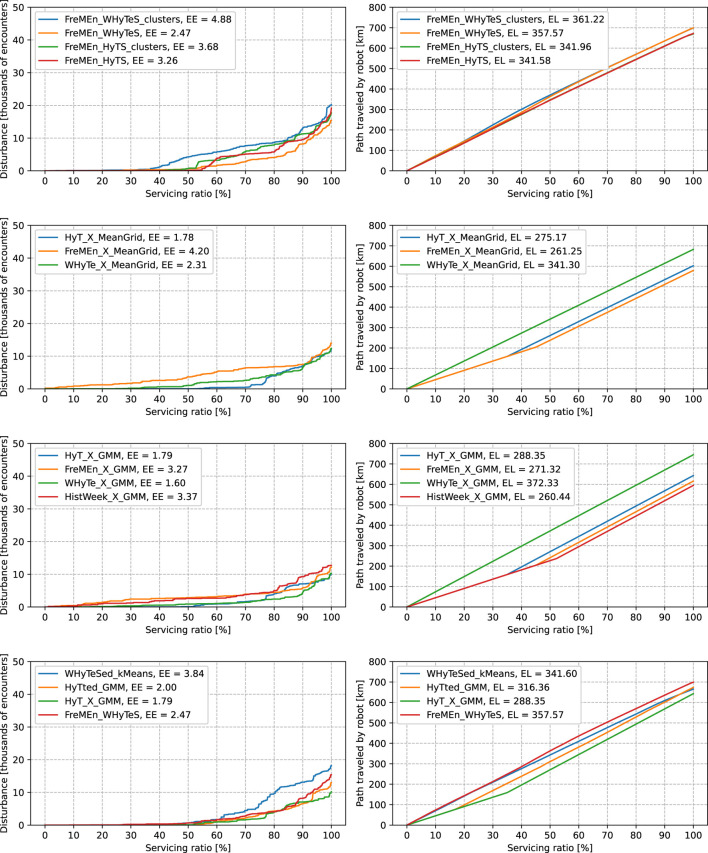
Comparison of different models in the spatio-temporal scenario. The left columns of graphs depict service disturbances, the quantile functions of the ordered human–robot encounters. The right columns of graphs depict traveled distances, the quantile functions of lengths of paths. The labels in graphs consist of names of models and the expected values.

In Figure 6, the top row provides a comparison of the continuous spatio-temporal models. All of them have a linear growth of the traveled distance, and neither can provide predictions that would let the robot travel by the shortest path. Moreover, the spatio-temporal model *FreMEn_WHyTeS_clusters* modeling five Gaussians performs worse in expected encounters than the similar model *FreMEn_WHyTeS* with only one component. The loss of quality between *FreMEn_HyTS_clusters* and *FreMEn_HyTS* is not so drastic. However, the evaluation of the continuous spatio-temporal models that apply *GMM* over the hypertime provides us with information that the robot always follows a long path. On the other hand, *FreMEn_WHyTeS* provides the best expected encounters between methods already compared.

The following two rows of graphs compare the spatio-temporal methods that model space and time separately as independent phenomena. Many of those methods perform surprisingly well, especially in choosing the right time to go by the shortest path. *HyT_X_MeanGrid* and *HyT_X_GMM* can lead the robot by the shortest path in 35% of the time while evading almost every encounter in 50% of the time. Other methods exchange the performance in the length of the path for safety. *FreMEn_X_MeanGrid*, *FreMEn_X_GMM*, and *HistWeek_X_GMM* were unable to evade encounters.

Based on the results above, we combine the good qualities of the previously tested ones. As the *HyT_X_MeanGrid* and *HyT_X_GMM* perform very well in raw combinations of spatial and temporal models and extending *FreMEn_HyTS* with multiple components, *FreMEn_HyTS_clusters*, did not result in a positive change of the quality, we combined *HyT* and *GMM* in a way that every component has its own temporal model. The method is denoted as *HyTted_GMM*. A similar process led us to create *WHyTened_kMeans*. *FreMEn_WHyTeS* performed quite well in the sense of expected encounters but did not find the shortest path. This behavior led to the idea of “natural” divisions of space by k-means clustering while applying the successful spatio-temporal method to the subsets. The comparison of these two models with *HyT_X_GMM* and *FreMEn_WHyTeS* can be seen in graphs in the bottom row of Figure 6. We can see that the disturbance distribution functions of *HyT_X_GMM*, *FreMEn_WHyTeS*, and *HyTted_GMM* are very similar. Out of these three models, *HyT_X_GMM* provides the best expected encounters, and *FreMEn_WHyTeS* the worst. *WHyTened_kMeans* performs much worse than others, and it did not even get the ability to provide the robot predictions that let it go through the environment with the shortest path. *HyTted_GMM* acquired the ability to predict short and safe paths more than 15% of the times while being a fully continuous spatio-temporal method.

**FIGURE 7:**
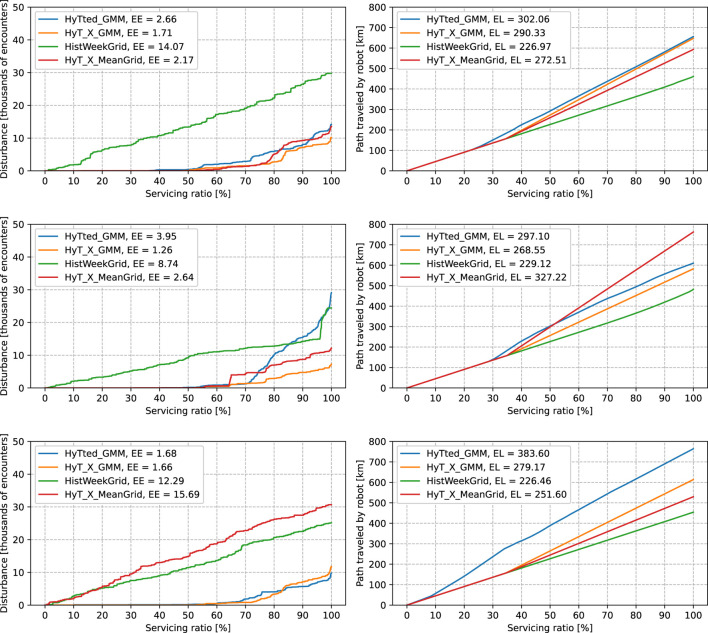
Comparison of selected models under different scenarios. The left columns of graphs depict service disturbances, the quantile functions of the ordered human–robot encounters. The right columns of graphs depict traveled distances, the quantile functions of lengths of paths. The labels in graphs consist of names of models and the expected values. First row: models trained over the sparse, spatio-temporal data. Second row: models trained over the spatio-temporal-directional data. Third row: models trained over the sparse, spatio-temporal-directional data.

We choose the three best-performing methods for the comparison in the subsequent scenarios, *HyT_X_GMM*, *HyT_X_MeanGrid*, and *HyTted_GMM*, together with *HistWeekGrid* which provided the best ability to lead the robot by the shortest path while performing exceptionally in evading encounters. On the top row of Figure 7, we provided a comparison of chosen methods in the spatio-temporal scenario when we randomly selected only one per mile of human detections from the training dataset to train the models. The *HistWeekGrid* lost its ability to provide predictions. In the previous scenario, predictions in each cell were calculated from approximately 2000 values, while in this scenario, they were calculated only from 2. The other three methods performed well. The *HyTted_GMM* is the worst of the rest of the methods in the range of 50, −,85%, but it can choose 25% of the safe and short paths, which is almost similar to the other two functional models. Moreover, *HyTted_GMM* and *HyT_X_MeanGrid* found shorter “safe” paths than *HyTted_GMM*, which can be deduced from different inclinations of the traveled distances.

In the second row of graphs in Figure 7, we compare the same four methods in a spatio-temporal-directional scenario with the full amount of detections. The methods predict when to go, what path to choose, and what direction is the best to go with the flow of people. The *HistWeekGrid* provides us with an inferior model that can model only the most frequent parts of the spatio-temporal-directional grid. The *HyT_X_GMM* and *HyTted_GMM* perform similarly up to a servicing ratio of 70%, where *HyTted_GMM* chooses a more risky and shorter path to follow. After following the shortest path, the *HyT_X_MeanGrid* chooses the longest path out of the three well-performing models, which means it led the robot in close vicinity to walls. The best performance by means of evading people is provided by *HyT_X_GMM*.

In the bottom row, we can see the performance of the methods when providing predictions in the spatio-temporal-directional scenario obtaining only one per mile of samples for the training. The grid-based methods *HistWeekGrid* and *HyT_X_MeanGrid* were not able to provide a prediction. The continuous models perform similarly in expected encounters but differ mainly in the paths they choose. The *HyT_X_GMM* retains its behavior from the previous scenario. However, *HyTted_GMM* started with the shortest path in the first 10% of planned services; then, it found out encounters can emerge and reacted to that ‘hysterically,’ forcing the robot to go around the walls. At about 30% of the servicing ratio, it started to follow similar paths to *HyT_X_GMM*.

#### 4.2.6 Comparison of Approaches Using Popular Methods

For the sake of comprehensiveness, we compare the approaches to spatio-temporal mapping using measures of quality that appeared in the referenced literature (Section 2.5). The most favored measures of the quality of maps are mean square error, rooted mean square error (RMSE), mean negative log-likelihood, and mean log-likelihood (MLL). We choose RMSE and MLL as their representatives in the comparison. We also included the area under a receiver operating characteristic curve (AUC) (Hanley and McNeil, 1982) as a complex representative of popular measures derived from the confusion matrix. We also included chi-Square Distance (*χ* ^2^d) (Dodge, 2008), which appeared in articles in the last few years as an alternative to RMSE. Some measures of the quality of maps appeared in the literature very rarely. Many of them are hard to implement, and a few are poorly defined. We included the Pearson correlation coefficient (PCC) (Bravais, 1844) as a representative of rare measurements because we found it the most traditional and feasible to implement.

Table 2 provides the results of *χ* ^2^d, AUC, RMSE, MLL, and PCC. The slight differences between the values representing the quality of the models led us to compare them with high precision representations. The best spatio-temporal map is OccupancyGrid according to *χ* ^2^d, RMSE, and MLL. We interpret the result so that the detection of humans is quite a rare event. Therefore, the best predictor is the one that predicts almost zero probability everywhere. Other maps also consist of small valued predictions but higher than ‘almost zero.’ As the detection is one point in space and time, the (hypothetically correct) predictions in the vicinity of the detections raise the error of the whole model. Similarly, the output of PCC for all the approaches gave us almost zero correlation between maps and the detections. It is not reasonable to deduce any conclusions from such small values. Contrary to previous ones, the AUC provided a very reasonable ranking. We can deduce which of the approaches gave us good predictions. However, the interpretability of the outputs is very limited.

**TABLE 2:** Comparison of the approaches using popular metrics.

	*χ* ^2^d	AUC	RMSE	MLL	PCC
Section 4.2.3.1					
MeanGrid	1.319811e+06	0.678204	1.131719e-07	−5.964167e-07	0.004277
OccupancyGrid	**1.077451e+05**	0.500000	**1.107762e-07**	**−5.714333e-07**	0.000000
GMM	1.895740e+06	0.661219	1.119760e-07	−5.838786e-07	0.006606
Section 4.2.3.2					
FreMEn	1.088325e+06	0.708411	1.112586e-07	−5.764210e-07	0.017761
HyT	1.157291e+06	0.786832	1.115330e-07	−5.792680e-07	0.018118
WHyTe	1.896955e+06	0.735896	1.128428e-07	−5.929532e-07	0.021518
HistWeek	9.583215e+05	0.770708	1.112834e-07	−5.766781e-07	-0.010873
Section 4.2.3.3					
FreMEnGrid	7.824628e+05	0.724162	1.113846e-07	−5.777281e-07	0.019364
HistWeekGrid	4.436296e+05	0.790896	1.109813e-07	−5.735518e-07	-0.019459
time_window_GMM	8.572676e+05	0.811935	1.256092e-07	−7.347102e-07	0.014281
Section 4.2.3.4					
HistWeek_X_GMM	9.599045e+05	0.817996	1.112969e-07	−5.768189e-07	-0.011000
FreMEn_X_MeanGrid	7.703107e+05	0.745279	1.112063e-07	−5.758796e-07	0.006380
HyT_X_MeanGrid	8.180353e+05	0.816573	1.112772e-07	−5.766142e-07	0.005943
WHyTe_X_MeanGrid	1.318568e+06	0.768864	1.117257e-07	−5.812720e-07	0.009677
FreMEn_X_GMM	1.088325e+06	0.739453	1.111231e-07	−5.750178e-07	0.015433
HyT_X_GMM	1.154622e+06	**0.829502**	1.112700e-07	−5.765397e-07	0.013693
WHyTe_X_GMM	1.895740e+06	0.774540	1.141287e-07	−6.065450e-07	0.024228
Section 4.2.3.5					
FreMEn_HyTS_clusters	1.578800e+06	0.808032	1.121873e-07	−5.860852e-07	0.023457
FreMEn_WHyTeS_clusters	1.857979e+06	0.788587	1.134001e-07	−5.988245e-07	0.021970
FreMEn_HyTS	1.896951e+06	0.814097	1.140148e-07	−6.053347e-07	**0.032425**
FreMEn_WHyTeS	1.896950e+06	0.772722	1.135045e-07	−5.999280e-07	0.027456
Section 4.2.3.6					
HyTted_GMM	1.152000e+06	0.817629	1.113172e-07	−5.770285e-07	0.013649
WHyTened_kMeans	1.870547e+06	0.783438	1.136826e-07	−6.018122e-07	0.026585

The bold values highlight the best map for each metric.

We also included the correlation between results of all the metrics, Table 3. RMSE and MLL are highly correlated, providing almost interchangeable information. There is also a rather significant correlation between *χ* ^2^ *d* and PCC. The correlation between the AUC and other techniques is insignificant.

**TABLE 3:** Correlation of the popular metrics’ results.

	*χ* ^2^d	AUC	RMSE	MLL	PCC
*χ* ^2^d	1.00	0.29	0.14	−0.13	0.68
AUC	0.29	1.00	0.22	−0.21	0.18
RMSE	0.14	0.22	1.00	**−1.00**	0.26
MLL	−0.13	−0.21	**−1.00**	1.00	−0.25
PCC	0.68	0.18	0.26	−0.25	1.00

The bold values highlight the most correlated metrics.

## 5 Conclusion

Derived from our experiences with long-term deployments of autonomous robots, we hypothesize that respecting essential human habits improved robot efficiency and acceptability in the long term. In this study, we narrowed down this hypothesis for the case of human-aware navigation. We showed that a mobile robot which takes into account pedestrian flows and their changes over time is considered less disturbing. Our experiments indicated that the periodic properties of the flows allow for forecasting them even months after they were learned. A mobile robot using appropriate spatio-temporal maps can plan ahead to avoid situations where its navigation would disrupt the movement of people.

Therefore, we studied spatio-temporal modeling methods and provided a comprehensive overview. As those methods came from different fields and were intended for different usages, it was impossible to compare their performance in human-aware navigation. Derived from previous works, we proposed a methodology for defining a diverse collection of criteria for evaluating spatio-temporal maps. Those maps can be compared in their ability to provide beneficial forecasts for long-term deployments of human-aware autonomous robots.

We divided known approaches in a spatio-temporal mapping suitable to support long-term human-aware navigation, defined criteria for their comparison following the proposed methodology, and compared them in a simulation built using real-world data. The results showed that time series forecasting methods could not support path planning, but they can be used to avoid peak times. The spatial-only maps allow us to construct paths that avoid potentially crowded areas, but these paths are unnecessarily long in times when people are unlikely to be present. Discrete spatio-temporal maps can provide good predictions but require a large amount of data to achieve reasonable granularity. They are also memory inefficient and not scalable enough to be deployed in large environments. Continuous spatio-temporal maps modeling space and time together suffer from high computational complexity. On the other hand, continuous maps that modeled space and time independently were scalable, computationally efficient, and provided good predictions.

In this work, we presented a methodology that can assess the ability of spatio-temporal maps to support human-aware navigation and planning of service robots. These maps represent environment dynamics induced by human activities and habits. Using such a map to plan the paths the robot is supposed to traverse allows the robot to better blend into human-populated environments and evade socially inappropriate situations. We reviewed spatio-temporal map–building approaches and discussed their ability to support human-aware navigation.

## Data Availability

The datasets presented in this study can be found in online repositories. The names of the repository/repositories and accession number(s) can be found below: https://git.chronorobotics.tk/papers-public-implementations/2022_human_flows_experiments.
